# The Role of Percent Volume Buried in the Characterization of Copper(I) Complexes for Lighting Purposes

**DOI:** 10.3390/molecules25112647

**Published:** 2020-06-06

**Authors:** Murat Alkan-Zambada, Edwin C. Constable, Catherine E. Housecroft

**Affiliations:** 1 Laboratory of Inorganic Synthesis and Catalysis, Institute of Chemical Sciences and Engineering, École Polytechnique Fédérale de Lausanne (EPFL), ISIC-LSCI, BCH 3305, 1015 Lausanne, Switzerland; 2 Department of Chemistry, University of Basel, BPR 1096, Mattenstrasse 24a, CH-4058 Basel, Switzerland; edwin.constable@unibas.ch (E.C.C.); catherine.housecroft@unibas.ch (C.E.H.)

**Keywords:** copper(I), photoluminescence quantum yield, crystal structure, percent volume buried (%V_bur_), empirical correlations

## Abstract

The usefulness of percent volume buried (%V_bur_) as a readily quantifiable property is investigated with regard to [Cu(NN)(PP)]^+^ complexes of interest for lighting purposes. Photoluminescence quantum yields (PLQYs) and single crystal X-ray structures of 100 reported compounds were assembled, %V_bur_ of the ligand systems were calculated and analyzed for correlations. We found that increased shielding of the central Cu(I) cation relying on shared contributions of both (NN) and (PP) ligand systems led to increased PLQYs. These findings are of relevance for future characterizations of Cu(I)-based complexes and their photophysical behavior in the solid-state.

## 1. Introduction

Ionic transition metal complexes (iTMCs) are widely present in chemistry for e.g., catalysis or lighting purposes. Understanding of the electronic and steric effects of ligands on the iTMCs is essential to improve rational design of iTMCs for their varied purposes. While quantifying electronic effects of ligands onto metal complexes can be performed by spectroscopic methods such as IR, [1] similar quantification of the steric impact is less straight-forward. Tolman cone-angles have been introduced for the calculation of steric parameters [2] and widely used but suffer limitations as its usefulness is greatly diminished when applied to more elaborate mono- or bi-dentate ligands. Consequently, Nolan and Cavallo introduced the percent volume buried (%V_bur_) as a quantifiable parameter to describe any given ligand [3]. The %V_bur_ describes the volume occupied by any given ligand inside a sphere with a 3.5 Å radius around the metal center and topographical steric maps [4] are created using SambVca 2.1 package [5]. Since its introduction, the %V_bur_ parameter has proven to be useful as a descriptor of iTMCs in catalysis [6,7,8,9]. In the field of iTMC-based lighting devices, however, it has not gained attention despite its potential in structural analysis.

Heteroleptic copper(I)-based iTMCs have been employed in light-emitting electrochemical cells (LECs) and represent a promising avenue towards cost-efficient and sustainable lighting devices [10,11,12]. Consequently, understanding of the photophysical behavior of these complexes has guided their improved design for applications in LECs [13,14,15,16,17,18]. Electronic properties such as redox potentials, absorption and emission spectra are among the descriptors used in the analysis of Cu(I) complexes. Quantifiable descriptors of steric factors, however, are mostly limited to discussions regarding angles and/or bond-lengths in X-ray single crystal structures. We have recently evaluated the effect that Cu···O distances in the {Cu(POP)} and {Cu(xantphos)} domains of [Cu(P^P)(N^N)][X] (POP = bis(2-(diphenylphosphanyl)phenyl)ether; xantphos = 4,5-bis(diphenylphosphanyl)-9,9-dimethyl-9*H*-xanthene) compounds have on solid-state photoluminescence quantum yield (PLQY) values [19]. Discussions regarding steric shielding of the copper(I) complex cation by the ligands are mostly limited to simplistic comparisons of steric bulk imposed by ligand-substituents [19,20,21,22,23,24,25,26,27]. Therefore, we sought to investigate the usefulness of %V_bur_ with regard to Cu(I)-based iTMCs in lighting devices. As a case study we chose [Cu(NN)(PP)]^+^ systems (where NN and PP refer to bidentate diimine and mono- or bi-dentate bisphosphane ligands) due to its popularity and photoluminescence quantum yields (PLQYs) because of the importance of large PLQY values for lighting purposes.

## 2. Results and Discussion

### 2.1. General Strategies

**Figure 1:**
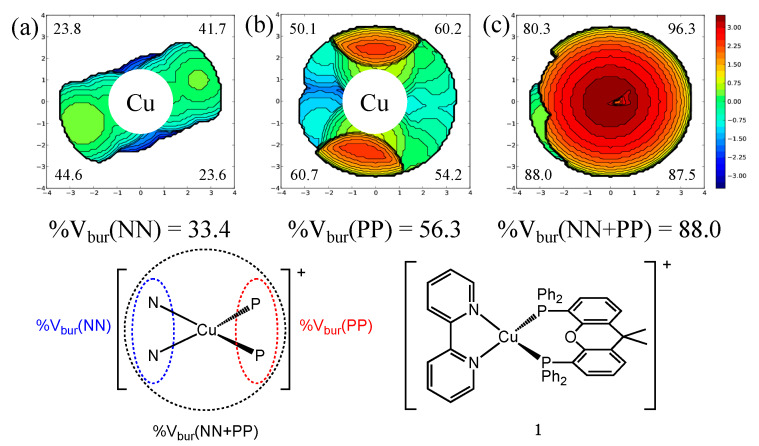
Topographical steric maps for bpy (**a**), xantphos (**b**) and for {(bpy)(xantphos)} (**c**) of [Cu(bpy)(xantphos)]^+^ (**1**) and a schematic representation of (NN), (PP) and (NN)(PP) subunits indicated in dotted lines used for the calculations of the respective percent volume buried (%V_bur_). Values in the corners of the topographical steric maps refer to the %V_bur_ of the respective quadrants and the total %V_bur_ are listed below. Axes of the topographical steric maps (in Å) refer to the X and Y reference axes and the Z axis is indicated by the contour lines (positive values refer to the upper hemisphere).

The Cambridge Structural Database (CSD) [28] was searched for compounds consisting of mononuclear [Cu(NN)(PP)]^+^ units where NN and PP refer to diimine and bisphosphane ligands. Searches were made using CSD version 5.4.1 and ConQuest version 2.0.4. [29]. The results were then further reduced to compounds with reported solid-state photoluminescence quantum yields (PLQYs). Particularly, values for thin-film PLQYs were not included as such values can differ from PLQYs of powder or crystal samples [11,18,25]. Moreover, PLQY values for crystalline samples were chosen over powder samples where such differentiations were made [30]. Similarly, PLQY values obtained under air were chosen over values obtained under an Ar atmosphere where such differentiations were made [31]. A total of 100 compounds were found and their %V_bur_ was calculated for the (NN), (PP) and (NN)(PP) units which are referred to as %V_bur_(NN), %V_bur_(PP) and %V_bur_(NN + PP) (Table S1, supporting information) [17,18,19,20,21,23,24,25,26,27,30,31,32,33,34,35,36,37,38,39,40,41,42,43,44,45,46,47,48]. All compounds contain bidentate diimine and bisphosphane ligands with one exception containing a bidentate diimine and two monodentate triphenylphosphano ligands (CCDC: 1558486) [45]. Where a compound contained more than one independent cation in the asymmetric unit, the %V_bur_ values were calculated for each independent cation, resulting in a total of 123 data points for 100 compounds. An inherent bias is present in these values as compounds with low PLQYs may be inevitably not fully investigated or reported with regards to solid state PLQY values and X–ray single crystal structures. Furthermore, the spread of %V_bur_ is biased by virtue of the commercial availability of ligands, the synthetic feasibility of ligand scaffolds as well as the synthetic feasibility of different [Cu(NN)(PP)]^+^ complexes. As the PLQY values were measured by different groups or by different means, those values may thus be subject to varying errors. Consequently, we assume an error of ±10% of a given PLQY value, e.g., ±5% for a PLQY of 50%. The end result of an analysis for a given compound is summarized in Figure 1 for the archetypical [Cu(bpy)(xantphos)]^+^ (**1**) (bpy = 2,2′-bipyridine). Topographical steric maps for the (NN), (PP) and (NN)(PP) subunits are shown including the total %V_bur_ as well as the %V_bur_ for the four quadrants. The topographical steric maps plot the metal at the center of the reference axes (X, Y) splitting the plot into four quadrants. The volume occupied by the ligands is indicated by contour lines (Z axis) where positive values refer to the upper hemisphere of the sphere.

### 2.2. Macroscopic Level

**Figure 2:**
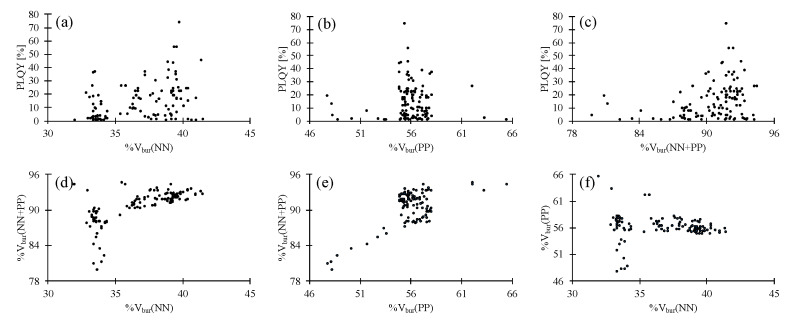
Scatter plots of photoluminescence quantum yield (PLQY), %V_bur_(NN), %V_bur_(PP) and %V_bur_(NN + PP) for all 100 compounds [17,18,19,20,21,23,24,25,26,27,30,31,32,33,34,35,36,37,38,39,40,41,42,43,44,45,46,47,48]. (**a**) Scatter plot of PLQY vs. %V_bur_(NN). (**b**) Scatter plot of PLQY vs. %V_bur_(PP). (**c**) Scatter plot of PLQY vs. %V_bur_(NN + PP). (**d**) Scatter plot of %V_bur_(NN) vs. %V_bur_(NN + PP). (**e**) Scatter plot of %V_bur_(PP) vs. %V_bur_(NN + PP). (**f**) Scatter plot of %V_bur_(NN) vs. %V_bur_(PP).

Scatter plots of the results for all 123 data points of the 100 compounds are shown in Figure 2. Careful analysis of these scatter plots suggests that, firstly, there is a small positive impact of increased %V_bur_(NN) on the PLQY (Figure 2a), and, second, an optimal %V_bur_(PP) seems to be located at ca 56%. An increase or decrease of %V_bur_(PP) negatively impacts the PLQY (Figure 2b). Thirdly, similar to %V_bur_(PP), there seems to be an optimal %V_bur_(NN + PP) located at ca 92% (Figure 2c). The fourth point is that %V_bur_(NN + PP) strongly depends on %V_bur_(PP) while the %V_bur_(NN) has little impact (Figure 2d–f).

**Figure 3:**
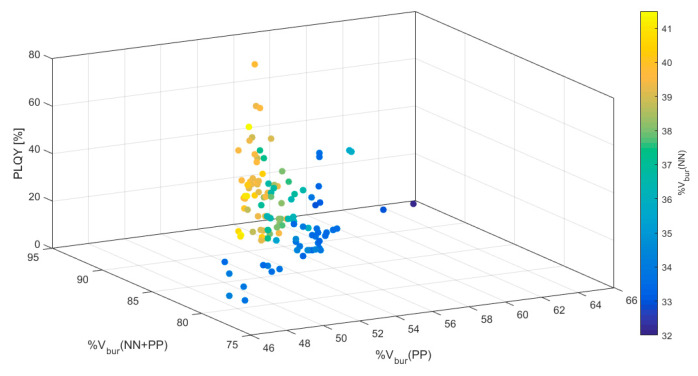
3-Dimensional scatter plot with color-coded %V_bur_(NN) of all 100 compounds found in the Cambridge Structural Database (CSD) (Table S1, Supplementary Materials) [17,18,19,20,21,23,24,25,26,27,30,31,32,33,34,35,36,37,38,39,40,41,42,43,44,45,46,47,48].

For a more detailed analysis of the results, color-coded three-dimensional scatter plots were generated including all four variables (Figure 3 and Figures S1–S3, Supplementary Materials). The PLQY was plotted against two %V_bur_ values with the remaining third %V_bur_ color-coded in the scatter plots. All three iterations of such plots support the dependence of large %V_bur_(NN + PP) for large PLQYs as seen in Figure 2c. Furthermore, these plots reveal that the best outcome is achieved by a shared %V_bur_ between the NN and PP ligands. A large %V_bur_(NN + PP) relying mostly on the PP ligand, on the other hand, yields low PLQYs. This shared contribution from both ligand units to the %V_bur_(NN + PP) seems to be the cause for the above mentioned observation regarding a perceived optimal %V_bur_(PP) at ca 56%. Most likely, a shared contribution of %V_bur_(NN) and %V_bur_(PP) within the [Cu(NN)(PP)]^+^ framework is more difficult to achieve for %V_bur_(PP) > 60% resulting in only three data-points in that regime. At this point it should be mentioned again that the values presented here are biased by synthetic feasibility and only serve to reflect empirical trends in reported values and the usefulness of %V_bur_.

### 2.3. Case Study 1: Importance of Steric Effects

**Figure 4:**
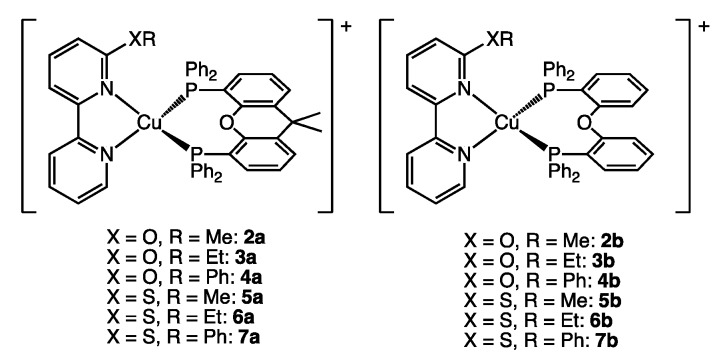
Structures of the [Cu(NN)(PP)]^+^ cations reported in reference [26] and used in case study 1.

In 2018, we reported twelve copper(I) complexes with 6-alkoxy- or 6-alkylthio-substituted bpy ligands in combination with xantphos (**2a**–**7a**) or POP (**2b**–**7b**) (Figure 4) [26]. The complexes were investigated by means of single crystal X-ray analysis, NMR spectroscopy, absorption and emission spectra, cyclic voltammetry as well as excited state lifetime measurements and density functional theory (DFT) calculations. The observed PLQY values (Table 1) were analyzed and a trend was established of increased PLQY by increased rigidity of xantphos compared to POP as well as increased steric bulk of the NN ligand in the order of bpyXMe < bpyXEt < bpyXPh (X = O, S). However, %V_bur_ is not included in this discussion and the explanation as such is only partially supported by the reported values (compare PLQYs of **2a** and **4a** or **2b** and **4b**). Thus, the available photophysical properties in combination with the relatively large set of substrates renders this set of compounds a suitable case study for the inclusion of %V_bur_ as a descriptor in the discussion of photophysical properties.

**Figure 5:**
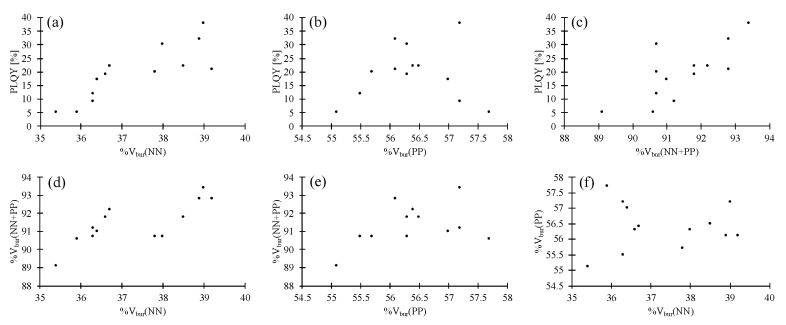
Scatter plots of PLQY, %V_bur_(NN), %V_bur_(PP) and %V_bur_(NN + PP) for compounds **2a/b**–**7a/b** [26]. (**a**) Scatter plot of PLQY vs. %V_bur_(NN). (**b**) Scatter plot of PLQY vs. %V_bur_(PP). (**c**) Scatter plot of PLQY vs. %V_bur_(NN + PP). (**d**) Scatter plot of %V_bur_(NN) vs. %V_bur_(NN + PP). (**e**) Scatter plot of %V_bur_(PP) vs. %V_bur_(NN + PP). (**f**) Scatter plot of %V_bur_(NN) vs. %V_bur_(PP).

The PLQY values for the series of complexes shown in Figure 4 as well as %V_bur_ are summarized in Table 1 and Figure 5. An analysis of the %V_bur_(NN) values shows that the intuitive assignment of increased steric bulk in the order of bpyXMe < bpyXEt < bpyXPh (× = O, S) is not reflected in the percent volume buried. Instead the following order is more appropriate based on the calculated %V_bur_(NN):

For P^P = Xantphos: bpyOPh < bpyOMe ≈ bpyOEt < bpySEt ≈ bpySPh ≈ bpySMe

For P^P = POP:    bpyOPh < bpyOMe ≈ bpyOEt < bpySPh ≈ bpySMe < bpySEt

**Figure 6:**
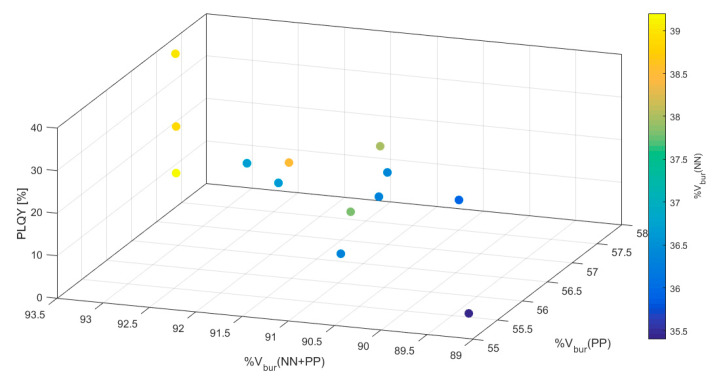
3-Dimensional scatter plot with color-coded %V_bur_(NN) for compounds **2a/b**–**7a/b** [26].

Overall, the %V_bur_(PP) values for POP are larger than those for xantphos with the exception of compounds **7a** and **7b**. Moreover, %V_bur_(NN + PP) seems to increase with %V_bur_(NN) while the largest %V_bur_(NN + PP) appears to be located at a medium %V_bur_(PP) of ca. 56.5%. Visualization of all four properties in color-coded three-dimensional scatter plots reveals that the highest PLQY values are reached when a high %V_bur_(NN + PP) is achieved by a combination of high %V_bur_(NN) and %V_bur_(PP) (Figure 6), in agreement with the interpretation of all 100 compounds (*vide supra*). Notably, comparing **5a** or **6a** to **7a** shows an increased PLQY with an increased %V_bur_(PP) or %V_bur_(NN + PP) while a similar correlation is not present when comparing **5a** to **6a** which only differ in %V_bur_(NN). Consequently, the nature of the differing PLQYs when comparing **5a** and **6a** is not due to the steric effects of the particular ligand system. Alternative explanations may be based on crystal packing such as improved accessibility of atmospheric O_2_ to the complex [20].

### 2.4. Case Study 2—Investigating Electronic Effects

**Figure 7:**
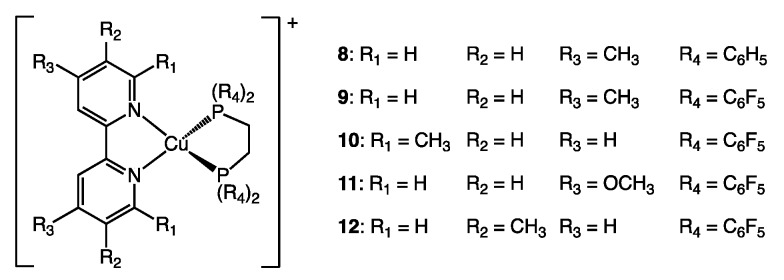
Structures of complexes **8–12** studied by Tsubomura and co-workers in 2015 [31].

Tsubomura and co-workers reported a series of seven heteroleptic copper(I) complexes in 2015 with the aim of investigating oxygen-responsive luminescence in the solid state. The single crystal X-ray structures of compounds **8**–**12** (Figure 7) were reported as well as powder PLQY values under air and under an argon atmosphere (Table 2) [31]. Given the choice of N^N and P^P ligands made by the authors, it is assumed that differences in PLQY are mostly due to electronic effects with the exception of **8** and **10**. The authors rationalize the trends in the observed PLQYs through differences in the crystal packing preventing structural rearrangement and differences in the transitions based on DFT calculations. Special emphasis was placed on the presence of voids in the crystal lattices which allows for excited state quenching by atmospheric O_2_. While significant differences in shielding by the ligands of the central copper(I) cation are not expected, we considered that an analysis of %V_bur_ may provide further insight. Most importantly, %V_bur_ values allow for a straight-forward, quantitative comparison of steric effects if these are indeed present.

**Figure 8:**
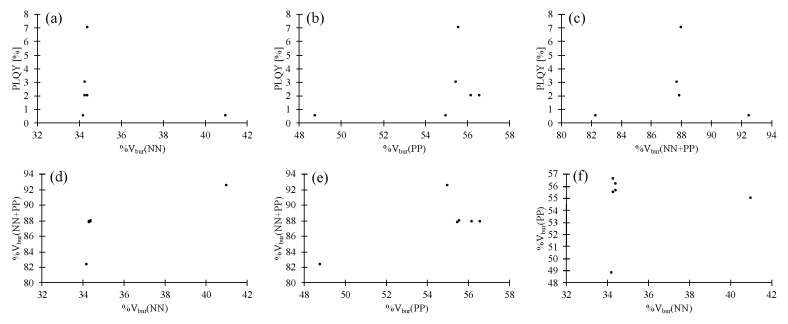
Scatter plots of PLQY, %V_bur_(NN), %V_bur_(PP) and %V_bur_(NN + PP) for compounds **8**–**12** [31]. (**a**) Scatter plot of PLQY vs. %V_bur_(NN). (**b**) Scatter plot of PLQY vs. %V_bur_(PP). (**c**) Scatter plot of PLQY vs. %V_bur_(NN + PP). (**d**) Scatter plot of %V_bur_(NN) vs. %V_bur_(NN + PP). (**e**) Scatter plot of %V_bur_(PP) vs. %V_bur_(NN + PP). (**f**) Scatter plot of %V_bur_(NN) vs. %V_bur_(PP).

An analysis of the scatter plots in Figure 8 indicates that steric effects may participate in the decrease of PLQY in the case of **8** and **10**. The remaining compounds are almost identical in the %V_bur_ values and any differences in PLQY may be attributed to electronic effects. On the other hand, effects due to differences in crystal packing can be excluded as changes in PLQY under air or under Ar atmosphere are absent or negligible. Only complex **10** exhibits a significant increase in PLQY upon removal of atmospheric O_2_. Structural shielding by the ligand system for compound **10** may be present but is negated by the presence of voids in the crystal packing and effective quenching of the excited state by O_2_ and energy transfer occurs instead. The lack of change in the PLQYs for the remaining compounds may be explained by structural rigidity inferred by the crystal packing rather than shielding of the central copper(I) cation.

### 2.5. Case Study 3—Differentiating Steric from Electronic Factors

**Figure 9:**
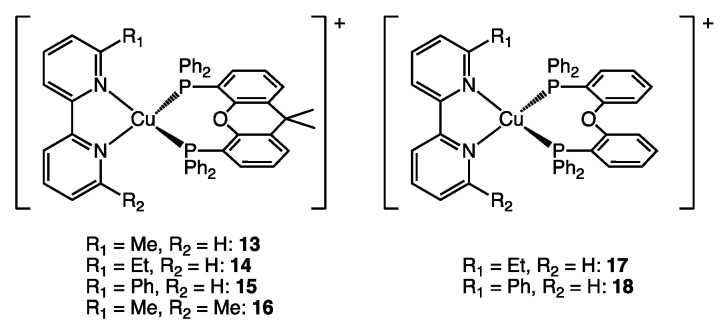
Structures of complexes **13**–**18** from reference [18].

In 2016, two of us reported a series of heteroleptic [Cu(NN)(PP)]^+^ complexes with 6- or 6,6’-substituted bpy ligands in combination with POP or xantphos (**13**–**18**, Figure 9) [18]. Following investigations of the structural and photophysical properties, the complexes were tested in LECs and were also investigated using DFT calculations. While high PLQY values were obtained for complexes **13**, **14**, **16** and **17** (Figure 9), it was observed that replacing the Me or Et substituent with a Ph group resulted in a significantly lower PLQY (Table 3). We ascribed this sharp drop in PLQY to a lower T_1_ state leading to increased feasibility of non-radiative decay pathways for **15** and **18**. We note that the structural discussions in the original publication did not provide a conclusive relationship between steric effects on the observed PLQYs. While %V_bur_ does not yield any insight into electronic effects, it does offer a concise overview of possible steric effects.

**Figure 10:**
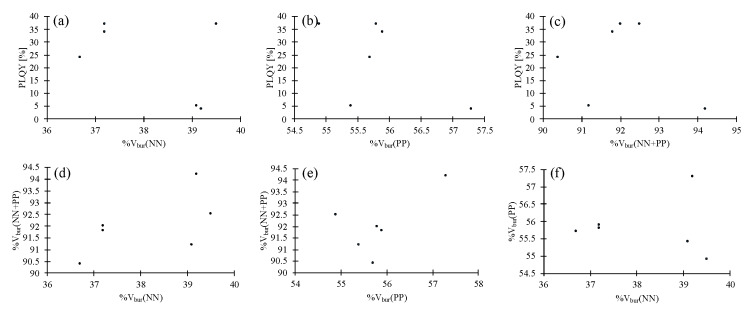
Scatter plots of PLQY, %V_bur_(NN), %V_bur_(PP) and %V_bur_(NN + PP) for compounds **13**–**18** [18]. (**a**) Scatter plot of PLQY vs. %V_bur_(NN). (**b**) Scatter plot of PLQY vs. %V_bur_(PP). (**c**) Scatter plot of PLQY vs. %V_bur_(NN + PP). (**d**) Scatter plot of %V_bur_(NN) vs. %V_bur_(NN + PP). (**e**) Scatter plot of %V_bur_(PP) vs. %V_bur_(NN + PP). (**f**) Scatter plot of %V_bur_(NN) vs. %V_bur_(PP).

**Figure 11:**
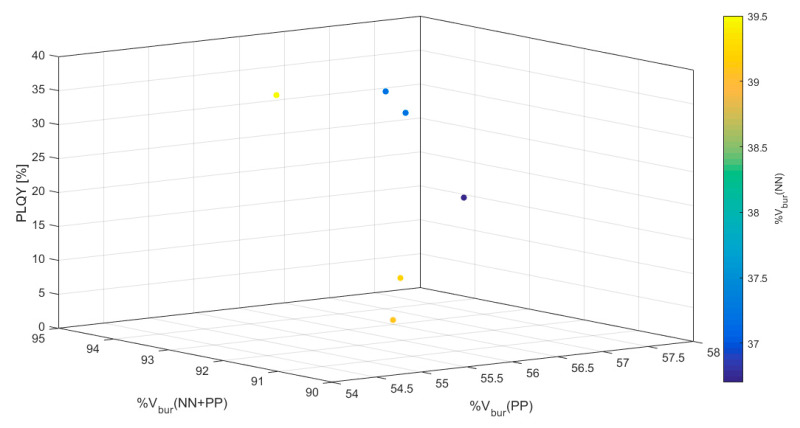
3-Dimensional scatter plot with color-coded %V_bur_(NN) for compounds **13**–**18** [18].

Analysis of the %V_bur_ values does not result in a clear correlation between the shielding of the central Cu(I) cation and PLQY values (Table 3). Graphical representation of these values further shows that the observed low PLQYs for **15** and **18** do not fit any possible correlation (Figure 10). Lastly, color-coded three-dimensional scatter plots clearly reveal that large PLQY values are obtained by virtually any combination of %V_bur_ with no correlation (Figure 11) as opposed to the correlations seen in case study 1 or in the analysis of all 100 complexes. This further supports the original hypothesis of lowered PLQY values due to a lowered T_1_ state by virtue of the phenyl substituent.

## 3. Materials and Methods

The Cambridge Structural Database (CSD) [28] was searched for compounds consisting of mononuclear [Cu(NN)(PP)]^+^. Searches were made using CSD version 5.4.1 and ConQuest version 2.0.4 [29]. Literature sources of the results were searched for reports of appropriate PLQY values which were collected together with the CIF files (Table S1 in the Supplementary Material). Topographical steric maps [4] were created using SambVca 2.1 package (https://www.molnac.unisa.it/OMtools/sambvca2.1/index.html) [5]. XYZ of the crystal structures were uploaded and the Cu(I) cation was defined as the center of the sphere. The negative Z-axis was defined by the N-atoms of the diimine for %V_bur_(NN) and %V_bur_(NN + PP) or by the P-atoms of the bisphosphane for %V_bur_(PP). The P-atoms of the bisphosphane defined the XZ-plane for %V_bur_(NN) and %V_bur_(NN + PP) and the N-atoms of the diimine for %V_bur_(PP). All atoms, except for the diimine ligand, bisphosphane ligand or both ligands, were deleted to calculate %V_bur_(NN), %V_bur_(PP) and %V_bur_(NN + PP), respectively. The Bondi radii were scaled by 1.17 and the sphere radius chosen as *r* = 3.5 Å. Mesh spacing of 0.10 Å was used for the numerical integration and H-atoms were excluded.

## 4. Conclusions

We have demonstrated that values of %V_bur_ represent a means of quantifying relationships between structure and PLQYs of heteroleptic [Cu(NN)(PP)]^+^ complexes. Using the CSD as a source of structural data, we were able to analyze 100 [Cu(NN)(PP)]^+^ complexes for which solid state PLQYs and single-crystal X-ray structures were available; this provided 123 data points. Analysis of all complexes showed that PLQY values are the highest when %V_bur_(NN + PP) is maximized by an increased %V_bur_(NN) and %V_bur_(PP). Three case studies underline the potential usefulness of including %V_bur_ as an addition in the structural discussion of [Cu(NN)(PP)]^+^ complexes as it relates to solid-state photophysical behavior.

## Figures and Tables

**Table 1:** Reported PLQY values for powder samples for Cu(I) complexes and %V_bur_ for (NN), (PP), and (NN)(PP) units. Data are from reference [26].

Complex	PLQY [%]	%V_bur_(NN)	%V_bur_(PP)	%V_bur_(NN + PP)
**2a**	19	36.6	56.3	91.8
**2b**	17	36.4	57.0	91.0
**3a**	22	36.7	56.4	92.2
**3b**	9	36.3	57.2	91.2
**4a**	12	36.3	55.5	90.7
**4b ^a^**	5	35.4/35.9	55.1/57.7	89.1/90.6
**5a**	21	39.2	56.1	92.8
**5b**	30	38.0	56.3	90.7
**6a**	32	38.9	56.1	92.8
**6b**	22	38.5	56.5	91.8
**7a**	38	39.0	57.2	93.4
**7b**	20	37.8	55.7	90.7

^a^ Two crystallographically independent cations are present in the asymmetric unit.

**Table 2:** Reported PLQY values for powder samples for copper(I) complexes and %V_bur_ for (NN), (PP), and (NN)(PP) units. Data are from reference [31].

Complex	PLQY [%]	%V_bur_(NN)	%V_bur_(PP)	%V_bur_(NN + PP)
**8**	0.5 (0.5) ^a^	34.2	48.8	82.3
**9**	7 (8) ^a^	34.4	55.6	88.0
**10**	0.5 (9) ^a^	41.0	55.0	92.5
**11**	3 (3) ^a^	34.3	55.5	87.7
**12 ^b^**	2 (2) ^a^	34.3/34.4	56.6/56.2	87.9/87.9

^a^ Value in parenthesis is for sample under Ar atmosphere. ^b^ Two crystallographically independent cations are present in the asymmetric unit.

**Table 3:** Reported PLQY values for powder samples for copper(I) complexes and %V_bur_ for (NN), (PP), and (NN)(PP) units. Data are from reference [18].

Complex	PLQY [%]	%V_bur_(NN)	%V_bur_(PP)	%V_bur_(NN + PP)
**13**	34	37.2	55.9	91.8
**14**	37	37.2	55.8	92.0
**15**	3.7	39.2	57.3	94.2
**16**	37	39.5	54.9	92.5
**17**	24	36.7	55.7	90.4
**18**	5.2	39.1	55.4	91.2

## Supplementary Materials

The following are available online: Figure S1–S3: 3-Dimensional scatter plot with color-coded %V_bur_(PP), %V_bur_(NN + PP) or PLQY of all 100 compounds found, Table S1: Reported PLQY values for powder samples for Cu(I) complexes and %V_bur_ for (NN), (PP), and (NN)(PP) units.
