# Supplementary material for: The Role of Percent Volume Buried in the Characterization of Copper(I) Complexes for Lighting Purposes
Source: Molecules. 2020 Jun 6;25(11):2647. doi: 10.3390/molecules25112647 (PMC7321245; doi:10.3390/molecules25112647)
Supplement: Supplementary file 1 [file molecules-25-02647-s001.pdf]

Supporting Material

The Role of Percent Volume buried in the Characterization of Copper(I) Complexes for Lighting Purposes

Murat Alkan-Zambada [1*], Edwin C. Constable [2] and Catherine E. Housecroft [2]

[1] Laboratory of Inorganic Synthesis and Catalysis, Institute of Chemical Sciences and Engineering, École Polytechnique Fédérale de Lausanne (EPFL), ISIC-LSCI, BCH 3305, Lausanne 1015, Switzerland; murat.alkan@epfl.ch

[2] Department of Chemistry, University of Basel, BPR 1096, Mattenstrasse 24a, CH-4058 Basel, Switzerland; Catherine.Housecroft@unibas.ch

* Correspondence: murat.alkan@epfl.ch

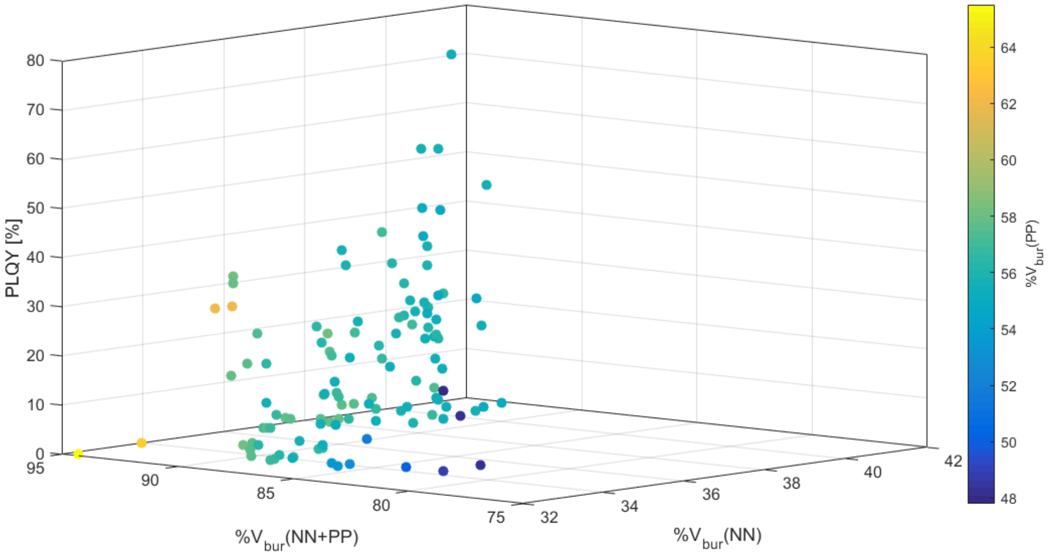

Figure S1. 3-Dimensional scatter plot with color-coded %V$_{bur}$*(PP) of all 100 compounds found.*

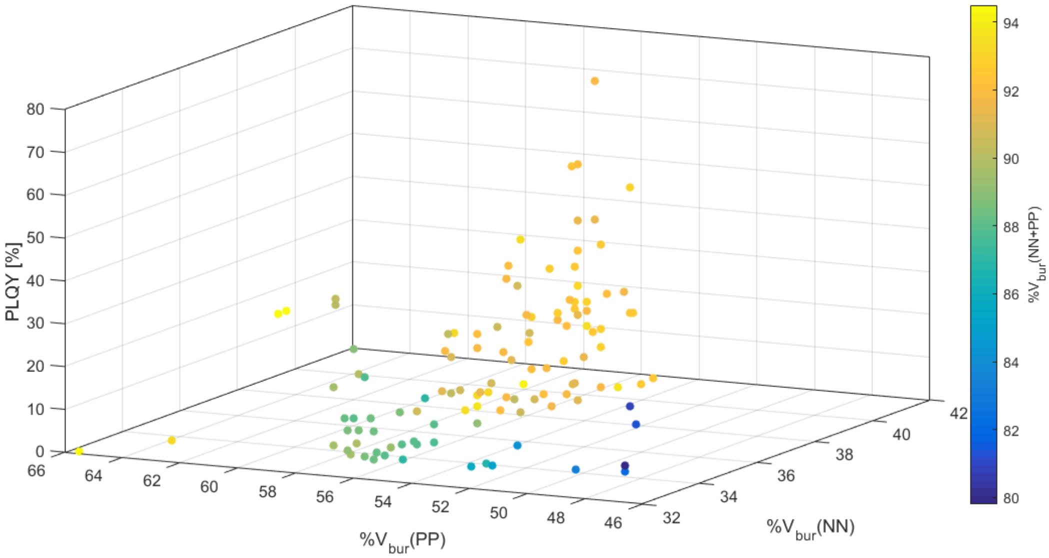

Figure S2. 3-Dimensional scatter plot with color-coded %V_{bur}(NN+PP) of all 100 compounds found.

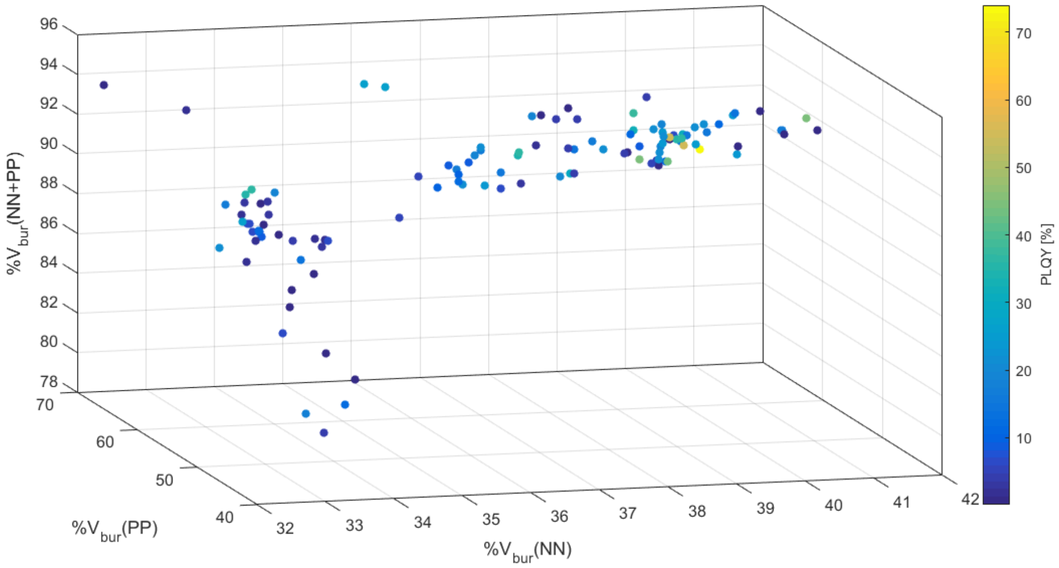

Figure S3. 3-Dimensional scatter plot with color-coded PLQYs of all 100 compounds found.

Table S1. Reported PLQY values for powder samples for Cu(I) complexes and %V_{bur} for (NN), (PP), and (NN)(PP) units.

Index	CSD Refcode and CCDC code		PLQY [%]	N^N [%]	P^P [%]	N^N+P^P [%]
1	UDOWOC	1562448	38	39.0	57.2	93.4
2	UDOXIX	1562457	12	36.3	55.5	90.7
3	UDOXUJ	1562460	32	38.9	56.1	92.8
4	UDOWIW	1562411	22	36.7	56.4	92.2
5	UDOXOD	1562458	21	39.2	56.1	92.8
6	UDOWAO	1562409	19	36.6	56.3	91.8
7	UDOXET	1562453	20	37.8	55.7	90.7
8[a]	UDOWUI	1562412	5	35.9	57.7	90.6

9[a]	UDOWUI	1562412	5	35.4	55.1	89.1
10	UDOVOB	1562407	22	38.5	56.5	91.8
11	UDOWES	1562410	9	36.3	57.2	91.2
12	UDOXAP	1562449	30	38.0	56.3	90.7
13	UDOVUH	1562408	17	36.4	57.0	91.0
14[a]	MEWXUK	1535142	17.1	41.0	55.4	92.6
15[a]	MEWXUK	1535142	17.1	41.0	55.3	92.6
16	MEWZAS	1583875	16.3	38.3	56.2	92.3
17[a]	MEWYEV	1535144	14.8	40.4	56.3	93.4
18[a]	MEWYEV	1535144	14.8	40.4	56.3	93.4
19	MEWXOE	1535141	3.9	38.0	56.7	91.9
20	GABTUB	1422372	34	37.2	55.9	91.8
21	GABWAK	1429456	37	37.2	55.8	92.0
22	GABMEE	1435492	3.7	39.2	57.3	94.2
23	GABVAJ	1422373	37	39.5	54.9	92.5
24	GABVEN	1422374	24	36.7	55.7	90.4
25	GABLAZ	1422375	5.2	39.1	55.4	91.2
26[a]	BOSVAI	996509	9.5	36.4	56.7	90.8
27[a]	BOSVAI	996509	9.5	36.1	56.7	90.2
28	BOSYUF	1009455	43.2	39.3	54.9	91.4
29	EVAFOY	1486911	44	38.9	55.1	91.5
30	EVAFEO	1486909	74	39.8	55.4	91.8
31[a]	EVADOW	1486906	11	39.3	55.3	91.3
32[a]	EVADOW	1486906	11	39.3	55.3	91.3
33	EVADUC	1486907	1	39.2	55.4	91.1
34[a]	EVAFAK	1486908	25	39.7	54.9	92.2
35[a]	EVAFAK	1486908	25	39.7	54.9	92.2
36	YITSIG	1844060	3	33.6	56.3	89.3
37	HIJQUP	1844062	1.9	33.0	63.3	93.3
38	HIJRAW	1844063	16	36.9	55.4	91.1
39[a]	YITSOM	1844061	26	35.5	62.1	94.5
40[a]	YITSOM	1844061	26	35.8	62.1	94.3
41	VICRAD	1581158	1.7	33.4	56.3	88.0
42[a]	VICQUW	1581155	11.1	40.1	55.5	93.0
43[a]	VICQUW	1581155	11.1	38.8	55.5	92.7
44	VICREH	1581157	0.9	33.8	57.0	88.1
45	OYUKID	802645	3	33.7	57.6	89.7
46	VICQOQ	1581154	6.2	39.6	57.4	92.2
47	VICROR	1581156	0.5	33.6	57.7	89.6
48[a]	VICRIL	1581159	0.5	33.6	57.2	88.6
49[a]	VICRIL	1581159	0.5	33.4	56.7	88.4
50	REQCIC	1583820	0.4	32.0	65.5	94.3
51	VAWFAD	1528552	4	38.0	55.7	90.8
52	VAWDOP	1528550	4.6	39.2	55.6	91.3

53	VAWDEF	1528548	0.3	38.8	55.9	91.7
54	VAWDUV	1528551	3.6	38.7	55.3	91.8
55	VAWDIJ	1528549	0.3	40.7	55.5	93.6
56	JUCFEV	1963589	35	39.5	55.7	92.3
57	JUCDUJ	1963587	19	37.6	58.1	93.3
58	ZUFVOO	1966895	45	41.4	55.8	93.1
59	ZUFVII	1966894	21	32.9	56.5	87.7
60	ZUFWAB	1966897	12	33.4	55.8	88.6
61[a]	ZUFWEF	1966898	24	40.3	54.9	91.6
62[a]	ZUFWEF	1966898	24	40.4	56.6	93.2
63[a]	ZUFVUU	1966896	3	33.4	58.1	89.6
64[a]	ZUFVUU	1966896	3	33.4	58.1	89.6
65[a]	ZUFWIJ	1966899	7	33.4	57.2	88.7
66[a]	ZUFWIJ	1966899	7	33.4	56.7	88.4
67	OHERAX	1987595	9	36.5	56.1	91.5
68	OHERIF	1987597	17	39.6	56.2	92.1
69	OHEREB	1987596	21	39.2	55.4	91.4
70	WOXHID	1958102	4	33.9	55.8	88.0
71	WOXHAV	1958100	7	36.9	55.3	90.3
72	WOXHEZ	1958101	3	37.2	55.4	90.5
73	WOXGUO	1958099	14	34.0	55.5	87.1
74[a]	FUFZUD	1041066	0.8	39.5	56.9	92.1
75[a]	FUFZUD	1041066	0.8	41.5	55.1	92.6
76	FUGBAM	1041067	0.8	40.4	55.8	91.8
77	VANYOB	1522132	18.9	33.8	57.6	90.1
78	VANYUH	1522133	9.7	33.5	56.9	88.3
79	VANZES	1522135	1.8	33.2	55.5	87.1
80	HONMII	978700	0.1	33.7	53.6	86.0
81	HONMOO	978701	0.1	34.0	53.4	86.8
82	RARYIU	826035	18.33	33.4	47.8	80.9
83	RARYOA	826036	3.58	33.7	48.3	79.8
84	TUHXAY	1907394	55	39.6	55.8	92.0
85	TUHXEC	1907395	17	36.8	57.6	91.8
86	UHINAC	1042034	0.5	34.2	48.8	82.3
87	UHINIK	1042035	7	34.4	55.6	88
88	UHINUW	1042036	0.5	41.0	55.0	92.5
89	UHIPEI	1042037	3	34.3	55.5	87.7
90[a]	UHIPIM	1042038	2	34.3	56.6	87.9
91[a]	UHIPIM	1042038	2	34.4	56.2	87.9
92	ZIQCUA	1864278	22	36.5	57.2	90.2
93[a]	FAJCIF	1422059	0.8	37.7	57.8	93.4
94[a]	FAJCIF	1422059	0.8	38.1	57.8	93.7
95[a]	CEYYIR	1588519	4.1	37.9	57.6	93.2
96[a]	CEYYIR	1588519	4.1	38.2	57.5	93.2

97	CEYYOX	1588520	2.2	37.5	56.4	92.2
98	LOHDEU	1558486	17.37	33.1	57.8	89.6
99	LOHFAS	1912912	6.91	33.4	51.7	84.2
100	LOHFIA	1912914	2.36	33.3	57.5	89.1
101	LOHFUM	1912915	35.74	33.4	58.0	90.0
102	LOHGAT	1912916	6.87	33.4	57.6	88.6
103	YOGCUV	1887568	37	33.5	58.1	90.2
104[a]	ARURIP	829666	19	39.3	56.2	91.9
105[a]	ARURIP	829666	19	39.4	57.2	92.8
106	ARUROV	829667	31	39.6	55.9	92.5
107	ARURUB	829668	8.4	39.0	56.3	91.9
108[a]	ARUSAI	829669	22	39.9	55.8	93.0
109[a]	ARUSAI	829669	22	39.3	55.6	92.5
110[a]	ARUSAI	829669	22	39.8	56.1	92.8
111[a]	ARUSAI	829669	22	39.2	55.1	91.8
112	UGANUO	1588141	14	38.1	56.9	91.8
113	COYHEF	989087	55	39.4	55.8	92.4
114	MULGIL	1046965	1	33.9	50.2	83.4
115	MULGOR	1046966	1	33.6	52.8	85.3
116	MULGUX	1046967	12.4	34.0	48.2	81.2
117[a]	MULHAE	1046968	9	33.6	57.9	88.1
118[a]	MULHAE	1046968	9	33.6	57.6	87.9
119	MULHEI	1046969	26.2	33.3	57.3	88.8
120[a]	-	1995549	24	39.3	55.8	92.7
121[a]	-	1995549	24	39.3	55.8	92.1
122[a]	-	1995548	16	39.9	55.3	92.7
123[a]	-	1995548	16	39.6	55.3	92.6

[a]More than one crystallographically independent cation present in the asymmetric unit.
